# Efficacy and safety of PD‐1 monoclonal antibody combined with interferon‐alpha 1b and anlotinib hydrochloride as the second‐line therapy in patients with unresectable advanced melanoma: A retrospective study

**DOI:** 10.1002/cam4.70087

**Published:** 2024-08-21

**Authors:** Bolun Zhao, Mengyu Zhang, Jingyi Tang, Daopei Zou, Fang Liu, Qiong Shi, Tianwen Gao, Chunying Li, Guannan Zhu

**Affiliations:** ^1^ Department of Dermatology, Xijing Hospital Fourth Military Medical University Xi'an China

**Keywords:** advanced melanoma, anlotinib, IFN‐α1b, PD‐1, second‐line therapy

## Abstract

**Background:**

Immune‐checkpoint inhibitors are now used more commonly in combination than monotherapy as the first‐line choice in patients with unresectable advanced melanoma. Nevertheless, for cases that progressed after the initial combination therapy, the subsequent regimen option can be very difficult. Herein, we reported the efficacy and safety of a triple combination regimen in Chinese unresectable advanced melanoma patients who had poor responses to the first‐line immune therapy.

**Methods:**

We reviewed the clinical profiles of patients diagnosed with stage IIIC‐IV melanoma between June 1, 2020, and September 30, 2023. The patients who failed the prior immune therapies and received anti‐PD‐1 mono antibody plus interferon(IFN)‐alpha 1b and anlotinib hydrochloride as the second‐line therapy were enrolled in the retrospective analysis. Additionally, we examined the exhaustion of T‐cells using mIHC staining in available tumor samples.

**Results:**

Fifty‐five patients were included in this study. The median follow‐up period was 13.6 months. The objective response rate evaluated by the investigators was 9.1%(1CR, 4PR). The disease control rate was 47.3%. The median overall survival was 17.6 months, and the median progression‐free survival was 2.8 months. The adverse events rate of any grade was 100%. Grade 3 or 4 irAEs were observed in 29.1% of cases. Multiplex immunohistochemical staining revealed an increased trend of TIM3 expression on tumor‐infiltrating T cells in patients without objective response.

**Conclusion:**

PD‐1 monoclonal antibody plus interferon‐alpha 1b plus anlotinib showed acceptable tolerability and anticancer benefits in Chinese metastatic melanoma patients as a second‐line therapy.

## 1 INTRODUCTION

Although immune checkpoint inhibitors (ICIs) have revolutionized the systemic strategy of cancer management including melanoma, the treatment resistance remains a great challenge that impairs their anti‐tumor efficacy. Current understanding of mechanisms affecting the response to ICIs includes the immunogenicity of tumors, neoantigen depletion, defective antigen presentation, PD‐L1 downregulation, and upregulation of inhibitory immune‐ checkpoints, etc.^1^, ^2^ In recent years, a growing number of studies have demonstrated that the treatment responses of ICIs can be significantly improved by combining with a second drug.^3^, ^4^, ^5^, ^6^ Previously, we observed a 32.8% objective response rate in a retrospective cohort of Chinese metastatic melanoma patients treated with PD‐1 antibody plus IFN‐α1b,^7^ which is higher than the response rate for either of the two drugs reported in former literature in similar cohorts. Nevertheless, the development of treatment resistance is still not uncommon in patients receiving ICI‐based combination therapies. Usually, the majority of refractory cases respond poorer on second‐line regimens, especially for those who do not carry targetable mutant genes.

Anlotinib is a novel oral multi‐targeting receptor tyrosine kinase inhibitor featured by selective inhibition of vascular endothelial growth factor receptor (VEGFR), fibroblast growth factor receptor (FGFR), platelet‐derived growth factor receptors (PDGFR), and c‐kit. It was approved by the China Food and Drug Administration (CFDA) to treat advanced solid tumors including non‐small‐cell lung cancer, soft tissue sarcoma, medullary thyroid carcinoma, etc. Also, it showed favorable efficacy and good tolerance in a broad range of malignancies in combination with immune checkpoint inhibitors.^3^, ^8^, ^9^, ^10^, ^11^

Therefore, we added anlotinib as a third drug to the combination of PD‐1 antibody and IFN‐α1b in advanced melanoma patients refractory to initial immune therapy and documented the treatment response as well as the toxicity of this regimen.

## 2 METHODS

### 2.1 Patients and study design

Profiles of patients with stage IIIC‐IV melanoma diagnosed in the Dermatology Department of Xijing Hospital from June 1, 2020 to September 30th, 2023 were reviewed. The inclusion criteria were: (i). unresectable advanced melanoma (stage IIIC‐IV, AJCC 8th Edition) confirmed by histological examination. (ii). Patients failed the first‐line immunotherapy (PD‐1 monotherapy or in combination with IFN‐α 1b). (iii). Patients receiving the triple combination of IFN‐α 1b (300ug or 600ug, subcutaneous injection, every other day) plus anti‐PD‐1 antibody (Pembrolizumab 200 mg/Toripalimab 240 mg/Sintilimab 200 mg, intravenous injection, every 3 weeks) plus anrotinib hydrochloride (oral, 12 mg daily for 14 days, discontinued for 7 days, every 21 days) as the second‐line therapy for a least 8 weeks. Patients with absent or incomplete clinical information were excluded.

The curative effect of solid tumors was evaluated by Response Evaluation Criteria in Solid Tumors (RECIST V1.1). Toxicity and grade were determined by Common Terminology Criteria for Adverse Events (CTCAE) V.5.0. This retrospective study was conducted by the Declaration of Helsinki. The Internal Review Board at the Air Force Military Medical University approved the project and waived the need for patient‐informed consent.

### 2.2 Statistical analyses

Continuous variables were reported as medians (ranges), while classified variables were reported as absolute frequencies and percentages. Kaplan–Meier curve is used to describe the progression‐free survival (PFS) and overall survival (OS). Univariate and multivariate Logistic regression analysis was used to analyze the factors related to objective response rate (ORR). Univariate and multivariate COX regression analysis was used to analyze the risk factors affecting PFS and OS. In the multivariate survival analysis, the variables (*p*‐value <0.10) that were significantly correlated with OS or PFS in the univariate analysis were introduced. In this paper, SPSS26.0 statistical software is used for statistical analysis. When *p* value<0.05, the difference is considered to be statistically significant.

### 2.3 Multiplex immunohistochemical (mIHC) staining and image analysis

Multiplex immunohistochemical (mIHC) staining was employed to detect the T‐cell exhaustion status in the tumor microenvironment of patients with and without treatment responses. CD8α‐70306 (Cell Signaling Technology (CST), Boston, USA), TIM3T‐45208 (CST), LAG3‐15372 (CST), PD1‐86163 (CST), and S100‐GZ031129 (Gene Tech, Shanghai, China) were used to obtain multiplex immunofluorescent staining. Multispectral imaging was also performed to aid in this process. The process involved the sequential application of distinct primary antibodies, incubation of secondary antibodies coated with horseradish peroxidase, and amplification of the tyramide signal. Following each TSA procedure, the slides underwent microwave heat treatment. After all human antigens had been labeled, nuclei were stained with 4′‐6′‐diamidino‐2‐phenylindole (DAPI, SIGMA‐ALDRICH). The Mantra System (PerkinElmer, Waltham, Massachusetts, US) was used to scan the stained slides to create multispectral images. This system records the fluorescent spectra at 20‐nm wavelength intervals from 420 to 720 nm with the same exposure time; the scans are then combined to create a single stack image. The spectrum of autofluorescence of tissues and each fluorescein was extracted from images of single‐stained and unstained sections, respectively. Moreover, the recovered images were utilized to create a spectrum library that the inForm image analysis software (PerkinElmer, Waltham, MA, USA) needed for multispectral unmixing. We were able to generate reconstructed images of sections that had been stripped of autofluorescence by using this spectrum library.

## 3 RESULTS

### 3.1 Baseline profiles

A total of 55 patients were included. Demographic characteristics and disease‐related profiles are presented in Table 1. The cohort consisted of 29 males (52.7%) and 26 females (47.3%). The median age was 58.0 years old (32.1–80.6 yo). The leading mutated gene was cKIT (*n* = 12/55, 21.8%), followed by NRAS (*n* = 8/55, 14.5%), while BRAF mutations were found in only 4 cases (7.3%). Half of the patients were negative for the above three gene mutations, and 3 (5.5%) patients's mutation data were unavailable. IV M1c accounted for the greatest proportion of the cohort with 30.9% (17/55), IIIC‐IIID and IV M1b accounted for the same proportion of 27.3% (15/55), IV M1a was 12.7% (7/55), and IV M1d was the least with only 3.6% (2/55). The acral subtype made up nearly a half of the enrolled cases (*n* = 27/55, 49.1%), the cutaneous subtype came second (*n* = 16/55, 29.1%) and the mucosal subtype was seen in 11/55 (20.0%). Twenty‐six patients (47.3%) had elevated LDH. At the initiation of the second‐line therapy, among the 55 patients, 53 had progressive disease, and 2 had stable disease in response to the front‐line treatment. As to the best response to the first‐line treatment of this cohort, 2 (3.6%) patients showed partial responses, 12 (21.8%) had stable disease, and 41 patients (74.5%) had progressive disease, suggesting that the studied cohort comprised patients with both initial and acquired resistance to PD1 + IFN.

**TABLE 1:** Baseline profiles.

	*n*	%
Sex		
Male	29	52.7
Female	26	47.3
Age		
≤60 years old	30	54.5
>60 years old	25	45.5
Median years	58.0	
Range	32.1–80.6	
Stage		
IIIC‐IIID	14	25.5
IV M1a	7	12.7
IV M1b	15	27.3
IV M1c	17	30.9
IV M1d	2	3.6
LDH		
Normal (≤ULN)	29	52.7
Elevated (>ULN)	26	47.3
ECOG PS		
0	45	81.8
≥1	10	18.2
Primary site		
Cutaneous	16	29.1
Acral	27	49.1
Mucosal	11	20.0
Unkown	1	1.8
Front line treatment		
PD‐1	2	3.6
PD‐1 + IFN	53	96.4
Overall response to front‐line treatment		
PD	53	96.4
SD	2	3.6
Best response to front‐line treatment		
PD	41	74.5
SD	12	21.8
PR	2	3.6
Gene mutation		
Wild type	28	50.9
BRAF	4	7.3
NRAS	8	14.5
cKIT	12	21.8
Unkown	3	5.5
PD‐1 monoantibody type		
Pembrolizumab	15	27.3
Toripalimab	38	69.1
Sintilimab	2	3.6
IFN dose		
600 μg	43	78.2
300 μg	12	21.8

### 3.2 Efficacy

**FIGURE 1:**
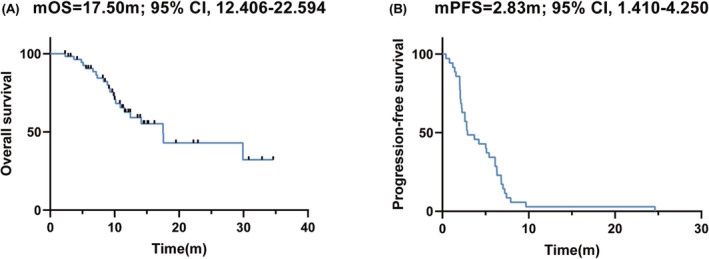
Kaplan–Meier curve for overall survival. (A) OS for patients. (B) PFS for patients. m: Month.

**FIGURE 2:**
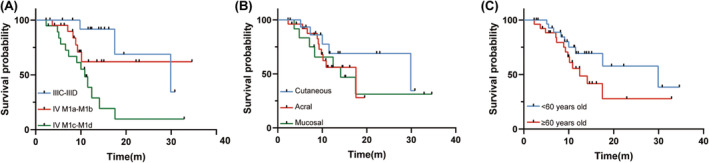
Kaplan–Meier curve for overall survival. (A) OS for patients with different Stage. *p* ^0^ = 0.005 (comparison between three groups); *p* ^1^ = 0.172 (IV M1a‐M1b vs. IIIC‐IIID); *p* ^2^ = 0.002 (IV M1c‐M1d vs. IIIC‐IIID). (B) OS for patients with different Primary site. *p* ^0^ = 0.730 (comparison between three groups); *p* ^1^ = 0.251 (Acral vs. Cutaneous); *p* ^2^ = 0.428 (Mucosal vs. Cutaneous). (C) OS for patients with different Age. *p* = 0.181 (<60 years old vs. ≥60 years old). m: month.

As of the data cutoff on December, 19th, 2023, the median follow‐up time was 13.6 months (95% CI 10.6–16.6 months). The median overall survival (mOS) was 17.6 months (95% CI 3.0–32.1 months) (Figure 1A)and the median progression‐free survival (mPFS) was 2.8 months (95% CI 1.1–4.5 months) (Figure 1B). The 6‐month PFS rate was 27.8% (95% CI 20.3%–35.3%). The 6‐month and 1‐year OS rate was 88.2% (95% CI 83.7–92.7%) and 58.1% (95% CI 50.2%–66.0%), respectively. By RECIST V1.1 assessment, only 1 (1.8%) patient achieved a complete response and 4 (7.3%) patients achieved a partial response, with an ORR of 9.1% and a disease control rate (DCR) of 47.3% (Table 2). The patient with complete response (CR) had acral melanoma, and of the 4 PR patients, 2 had acral melanoma, 1 had cutaneous melanoma, and 1 had mucosal melanoma. Patients with stage IIIC‐IIID showed a significantly longer mOS than patients with M1c‐M1d (29.9 months vs 10.9 months, *p* = 0.002, Figure 2A). Unexpectedly, the mOS of cutaneous melanoma did not differ significantly compared to that of either acral or mucosal subtypes (*p* = 0.251 and 0.428, Figure 2B). Also, the mOS in patients younger than 60 years old (29.9 months, 95% CI 7.2–52.6 months) was more than twice as the number in patients aged 60 years old or over (12.4 months, 95% CI 8.1–16.8 months), though the difference was not statistically significant (*p* = 0.181) (Figure 2C). Univariable Cox regression identified stage was the only factor associated with worse overall survival (IV M1a‐M1b: HR = 0.1, *p* = 0.009; IV M1c‐M1d: HR = 0.4, *p* = 0.059) (Table 3) while no significant association was found between PFS and other variables of interest (Table S1).

**TABLE 2:** Objective response to treatment assessed per RECIST v1.1, by investigators.

	*N*	%
CR	1	1.8
PR	4	7.3
SD	21	38.2
PD	29	52.7
CR + PR	5	9.1
CR + PR + SD	26	47.3

**TABLE 3:** Univariable and multivariable Cox regression for OS.

Variable	Univariable		Multivariable	
	HR(95%CI)	*p* value	HR(95% CI)	*p* value
Sex				
Female	1			
Male	1.402 (0.587–3.352)	0.447	—	—
Age				
≤60 years old	1			
>60 years old	1.800 (0.752–4.307)	0.187	—	—
Stage				
IIIC‐IIID	1			
IV M1a‐M1b	0.136 (0.031–0.609)	0.009	—	—
IV M1c‐M1d	0.392 (0.149–1.034)	0.059	—	—
LDH				
Normal (≤ULN)	1			
Elevated (>ULN)	1.268 (0.538–2.991)	0.587	—	—
ECOG PS				
0	1			
≥1	0.916 (0.266–3.155)	0.890	—	—
Primary site				
Cutaneous	1			
Acral	2176.068 (0.000–1.2118E97)	0.944	—	—
Mucosal	3550.811 (0.000–1.9751E97)	0.941	—	—
Unkown	3766.866 (0.000–2.0972E97)	0.940	—	—
Front‐line treatment				
PD‐1	1			
PD‐1 + IFN	21.053 (0.000–9092773.368)	0.645	—	—
Overall response to front‐line treatment				
PD	1			
SD	0.047 (0.000–14539.671)	0.636	—	—
Gene mutation				
Wild type	1			
BRAF	0.727 (0.161–3.291)	0.679	—	—
NRAS	0.430 (0.037–4.937)	0.498	—	—
cKIT	0.681 (0.112–4.153)	0.677	—	—
Unkown	0.473 (0.077–2.896)	0.418	—	—
PD‐1 monoantibody type				
Pembrolizumab	1			
Toripalimab	15236.164 (0.000–2.6666E133)	0.949	—	—
Sintilimab	30845.861 (0.000–5.393E133)	0.946	—	—
IFN dose				
600 μg	1			
300 μg	0.667 (0.223–1.994)	0.469	—	—

### 3.3 Safety profile

Fifty‐five enrolled patients all reported adverse events (AEs) of any grade, and 16 (29.1%) reported Grade 3 or 4 AEs. No treatment‐related deaths were observed. Eighteen adverse events occurred in more than 10% of the patients, including fever (*n* = 32, 58.2%), fatigue (*n* = 31, 56.4%), rash (*n* = 28, 50.9%), vitiligo (*n* = 26, 47.2%), decreased appetite (*n* = 22, 40.0%), hypothyroidism (*n* = 20, 36.4%), alanine/aspartate aminotransferase increased (*n* = 18, 32.7%), oral ulcer (*n* = 18, 32.7%), high blood pressure (*n* = 11, 20.0%), myalgia (*n* = 8, 14.5%), pruritus (*n* = 8, 14.5%), vomiting (*n* = 8, 14.5%), weight loss (*n* = 8, 14.5%), WBC count decrease (*n* = 8, 14.5%), hyperthyroidism (*n* = 8, 14.5%), joint pain arthralgia (*n* = 7, 12.7%), hemorrhage (*n* = 6, 10.9%) and hand‐foot syndrome(*n* = 6, 10.9%) (Table 4). These adverse events were introduced to regression models to examine the association with efficacy. We found patients who developed oral ulcers had a better ORR (OR = 12.3, 95% CI 1.4–104.1, *p* = 0.021) compared to those without oral ulcers (Table S2). Multivariate Cox regression analysis identified rash as the only factor favorable to better PFS (HR = 0.5, 95% CI 0.2–0.9, *p* = 0.033) and OS (HR = 0.4, 95% CI 0.1–0.9, *p* = 0.046) (Tables S3 and S4).

**TABLE 4:** Summary of treatment‐related irAEs.

	Any grade		≥Grade 3	
	*N*	%	*N*	%
Any	55	100	16	29.1
Fever	32	58.2	0	0
Fatigue	31	56.4	3	5.5
Rash	28	50.9	1	1.8
Vitiligo	26	47.2	0	0
Decreased appetite	22	40.0	3	5.5
Hypothyroidism	20	36.4	0	0
Oral ulcer	18	32.7	1	1.8
Alanine/aspartate aminotrans‐ferase increased	18	32.7	0	0
High blood pressure	11	20.0	0	0
Vomiting	8	14.5	2	3.6
Pruritus	8	14.5	0	0
Myalgia	8	14.5	0	0
Weight loss	8	14.5	0	0
WBC count decrease	8	14.5	0	0
Hyperthyroidism	8	14.5	0	0
Arthralgia	7	12.7	2	3.6
Hand‐foot syndrome	6	10.9	2	3.6
Hemorrhage	6	10.9	0	0
Diarrhea	4	7.3	0	0
Nausea	4	7.3	0	0
Lose hair or feathers	4	7.3	0	0
Dullness of the oral senses	3	5.5	1	1.8
Glucose increase	3	5.5	0	0
PLT decrease	2	3.6	0	0
Hypoadrenia	2	3.6	0	0
Triglycerides increased	2	3.6	0	0
Other mucosal adverse reactions	2	3.6	0	0
Headache	2	3.6	0	0
Autoimmune pneumonia	2	3.6	0	0
Myasthenia gravis	1	1.8	1	1.8
Guillain‐Barre syndrome	1	1.8	0	0
Pituitary inflammation	1	1.8	0	0
Elevated bilirubin	1	1.8	0	0
Cough	1	1.8	0	0
Shortness of breath	1	1.8	0	0
Hiss	1	1.8	0	0
Blister	1	1.8	0	0
Electrocardiographic abnormality	1	1.8	0	0
Nephritis	1	1.8	0	0
Reduced hemoglobin	1	1.8	0	0
Decreased albumin	1	1.8	0	0

### 3.4 T‐cell exhaustion markers

**FIGURE 3:**
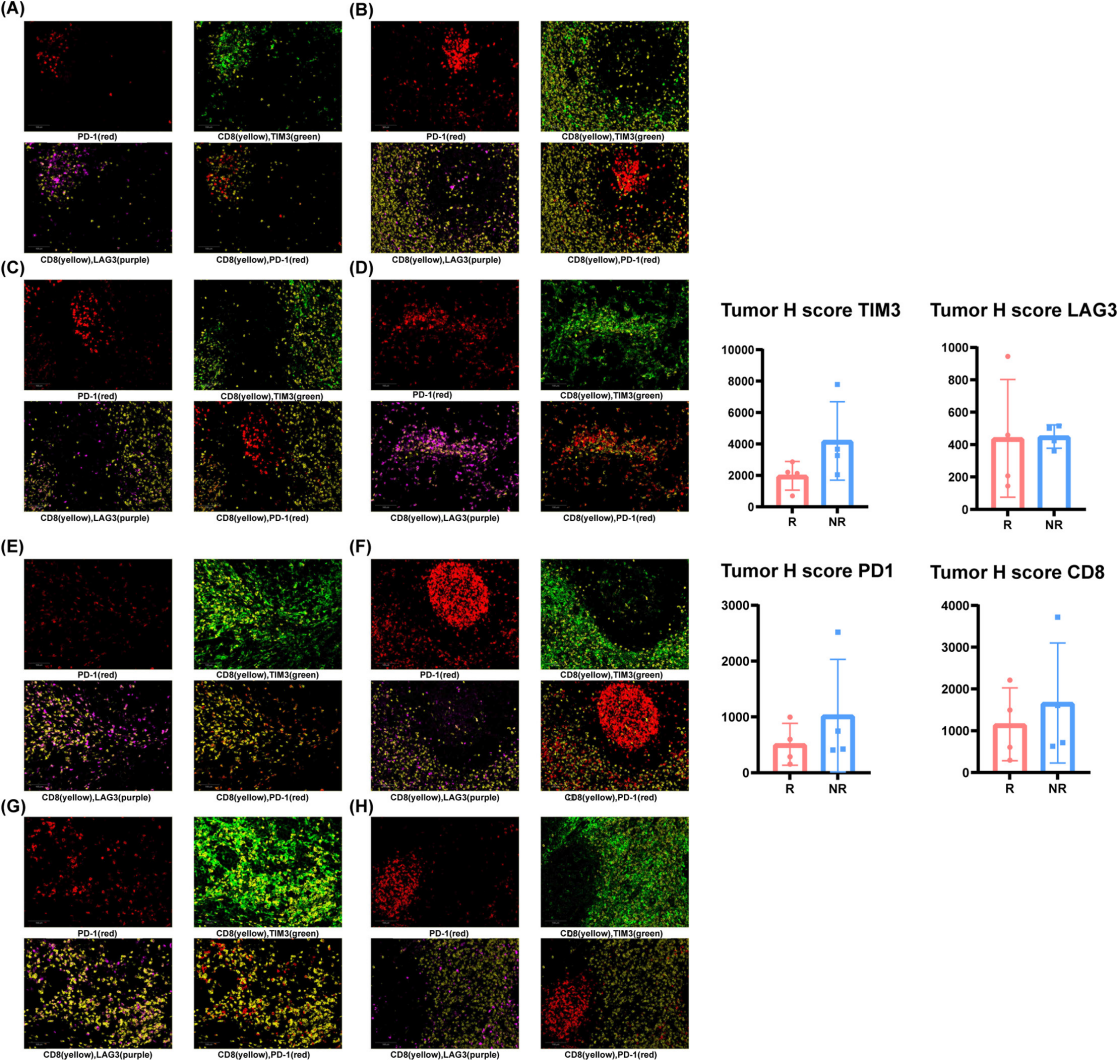
(A–D)Tumor tissue from patients with objective response to triple therapy for mIHC. (E–H) Tumor tissue from patients without objective response to triple therapy for mIHC. R, objective response; NR, no objective response.

To evaluate the impact of T‐cell exhaustion on the efficacy, we obtained 4 tumor samples from the individuals with treatment responses (1CR, 3PR) and 4 matched samples from those without treatment responses (1SD, 3PD). All samples were taken before the triple combination treatment was given. mIHC examination indicated a higher expression of TIM3 in patients without ORR. On the other hand, the expression of PD‐1, LAG‐3, and CD8 were similar between the two groups (Figure 3). Statistical calculation was not performed due to the small sample size.

## 4 DISCUSSION

Though the efficacy of immune‐checkpoint inhibitors in advanced melanoma has been improved by the combination of a second drug in most cases, the late‐line management for patients without treatment response on the initial treatment is a greater challenge. Novel strategies have been developed to overcome the resistance to ICIs by reprogramming the tumor microenvironment (e.g., combination of dual immune‐checkpoint inhibitors, adoptive T cell therapy, oncolytic treatments, cancer vaccines, etc.), for cases harboring specific pathogenic mutations, the addition of target inhibitors may bring synergetic benefits (e.g., BRAF and MEK inhibitors, PI3K inhibitors).^2^, ^12^, ^13^ In the current study, we performed a novel mixed regimen by adding anlotinib hydrochloride to a dual combination therapy of PD‐1 and IFN‐α1b which had previously been proven to have synergic efficacy in Chinese unresectable advanced melanoma patients.^14^

There were only five patients in the studied cohort who showed objective response on the triple combination regimen, yet 21 patients met stable disease criteria by RECIST V1.1, making the disease control rate 47.3% and mOS 17.6 months. Collectively, the triple combination of anti‐PD‐1 mono‐antibody plus IFNα1b and anlotinib hydrochloride showed some efficacy but no significant survival advantage than those reported in other late‐line regimens, such as Ipilimumab + Nivolumab,^15^, ^16^ lifileucel,^17^ apatinib^18^ and ceralasertib+ durvalumab (AZD6738),^19^ in advanced melanoma patients who had failed the prior immune therapies. It needs to be pointed out clearly that many characteristics may affect the results in the current studied cohort, including but not limited to the subtypes, stage distribution, driver mutation percentage, and the front‐line treatments. A majority of patients with BRAF mutation who failed the initial immune therapy in our center were prescribed dabrafenib + trametinib as the second‐line medication, making the extraordinarily low portion of BRAF mutation in our cohort.

Treatment‐related adverse events of any grade were reported in all patients (100%), but most of them were well‐tolerant, only 29.1% were grade 3/4 according to the CTC AE 5.0 definition. The most common events were fatigue, fever, and rash (all over 50%). Notably, oral ulceration was associated with ORR, and the rash was identified as a predictor of longer OS, suggesting a correlation between cutaneous irAE and better treatment efficacy.

To determine the biomarkers predicting the treatment response, we performed mIHC staining in available tumor samples from the cohort of patients. Among the 4 markers we examined, TIM3 is the only one that showed an upward trend in patients who did not respond to treatment compared to those who had responses. TIM3 has been proven to be a marker of T‐cell exhaustion and mediate the resistance to PD‐1 antibody.^20^, ^21^, ^22^, ^23^, ^24^, ^25^ However, we could hardly establish the correlation between TIM3 and the response of anlotinib addition due to the lack of control groups. In other words, the difference in TIM3 expression may reflect merely the response of the PD1 + IFN‐α1b combination as the second‐line therapy. However, these findings may suggest a reasonable use of TIM3 inhibitor as a late‐line option after the failure of PD‐1 + IFN‐α1b.

The limitations of this study include the small sample size and potential distribution bias due to the retrospective cohort from a single center. Also, the nature of the retrospective study posed great difficulty in sample collections, obstructing us from response‐related biomarker detection. Notably, excluding patients who received the combination therapy for less than 8 weeks may lead to potential selection bias. Moreover, the fact of sticking to the front‐line immune therapies may have largely excluded patients who may develop severe adverse events in response to the triple combination therapy. Some of the issues above will be addressed in our prospective trial to assess the efficacy and safety of the triple combination of IFN‐α1b + PD‐1 monoclonal antibody + anlotinib as the first‐line therapy in unresectable advanced melanoma(NCT05539118).

## 5 CONCLUSION

The triple combination of PD‐1 monoclonal antibody plus IFN‐α1b and anlotinib showed acceptable toxicity and certain anticancer efficacy as the second‐line therapy in Chinese advanced melanoma patients failed the prior immune therapies.

## AUTHOR CONTRIBUTIONS

**Bolun Zhao:** Data curation (equal); formal analysis (equal); investigation (equal); methodology (equal); software (equal); visualization (equal); writing – original draft (equal); writing – review and editing (equal). **Mengyu Zhang:** Data curation (equal); writing – original draft (supporting). **Jingyi Tang:** Data curation (equal); writing – original draft (equal). **Daopei Zou:** Data curation (equal); funding acquisition (equal); visualization (lead). **Fang Liu:** Conceptualization (equal); formal analysis (equal); methodology (equal). **Qiong Shi:** Funding acquisition (equal); methodology (equal); resources (equal). **Tianwen Gao:** Conceptualization (equal); formal analysis (equal); resources (lead). **Chunying Li:** Project administration (lead); supervision (lead). **Guannan Zhu:** Conceptualization (lead); data curation (lead); formal analysis (lead); funding acquisition (lead); investigation (lead); methodology (lead); project administration (equal); resources (equal); software (lead); validation (lead); writing – original draft (lead); writing – review and editing (lead).

## FUNDING INFORMATION

This work was supported by the National Natural Science Foundation of China (No. 82172607, 82273716, and 82103718).

## CONFLICT OF INTEREST STATEMENT

The authors declare no conflicts of interest.

## ETHICS STATEMENT

This study was approved by the Internal Review Board of Air Force Military Medical University (Fourth Military Medical University) (No. XJLL‐KY‐20232378‐C‐1).

## CONSENT

The Air Force Military Medical University's (Fourth Military Medical University) Internal Review Board waived the requirement for informed consent.

## Supporting information


Table S1.

## Data Availability

Upon reasonable request, the corresponding author will provide the data that support the study's conclusions.
