# Supplementary material for: Efficacy and safety of PD‐1 monoclonal antibody combined with interferon‐alpha 1b and anlotinib hydrochloride as the second‐line therapy in patients with unresectable advanced melanoma: A retrospective study
Source: Cancer Med. 2024 Aug 21;13(16):e70087. doi: 10.1002/cam4.70087 (PMC11337113; doi:10.1002/cam4.70087)
Supplement: Supplementary file 1 — Table S1. [file CAM4-13-e70087-s001.docx]

TABLE S1. Univariable and multivariable Cox regression for PFS

|  | Univariable | | Multivariable | |
| --- | --- | --- | --- | --- |
|  | HR(95%CI) | P value | HR(95%CI) | P value |
| Variable |  |  |  |  |
| **Sex** |  |  |  |  |
| Female | 1 |  |  |  |
| Male | 1.264(0.645-2.477) | 0.496 | — | — |
| **Age** |  |  |  |  |
| ≤60 years old | 1 |  |  |  |
| ＞60 years old | 0.936(0.477-1.836) | 0.847 | — | — |
| **Stage** |  |  |  |  |
| IIIC-IIID | 1 |  |  |  |
| IV M1a-M1b | 0.775(0.325-1.850) | 0.566 | — | — |
| IV M1c-M1d | 1.374(0.627-3.013) | 0.428 | — | — |
| **LDH** |  |  |  |  |
| Normal(≤ULN) | 1 |  |  |  |
| Elevated(＞ULN) | 1.138(0.583-2.220) | 0.705 | — | — |
| **ECOG PS** |  |  |  |  |
| 0 | 1 |  |  |  |
| ≥1 | 0.908(0.372-2.21) | 0.833 | — | — |
| **Primary site** |  |  |  |  |
| Cutaneous | 1 |  |  |  |
| Acral | 0.266(0.031-2.268) | 0.226 | — | — |
| Mucosal | 0.425(0.054-3.376) | 0.419 | — | — |
| Unkown | 0.393(0.046-3.387) | 0.396 | — | — |
| **Overall response to front-line treatment** |  |  |  |  |
| PD | 1 |  |  |  |
| SD | 0.340(0.045-2.560) | 0.295 | — | — |
| **Gene mutation** |  |  |  |  |
| Wild type | 1 |  |  |  |
| BRAF | 0.292(0.064-1.325) | 0.111 | — | — |
| NRAS | 0.710(0.063-7.973) | 0.781 | — | — |
| cKIT | 0.487(0.094-2.534) | 0.393 | — | — |
| Unkown | 0.218(0.042-1.125) | 0.069 | — | — |
| **PD-1 Monoantibody type** |  |  |  |  |
| Pembrolizumab | 1 |  |  |  |
| Toripalimab | 0.523(0.211-1.296) | 0.161 | — | — |
| **IFN dose** |  |  |  |  |
| 600μg | 1 |  |  |  |
| 300μg | 0.614(0.272-1.387) | 0.241 | — | — |

TABLE S2. Univariable and multivariable Logistic regression of irAE for ORR

|  | Univariable | | Multivariable | |
| --- | --- | --- | --- | --- |
|  | OR(95%CI) | P value | OR(95%CI) | P value |
| Fatigue | 1.525(0.309-7.526) | 0.604 | — | — |
| Fever | 3.143(0.328-30.158) | 0.321 | — | — |
| Rash | 1.500(0.230-9.763) | 0.671 | — | — |
| Vitiligo | 384636873.372(0.000-) | 0.998 | — | — |
| Decreased appetite | 1.510(0.363-6.290) | 0.571 | — | — |
| Hypothyroidism | 0.000(0.000-) | 0.999 | — | — |
| Oral ulcer | 12.281(1.449-104.051) | 0.021 | — | — |
| Alanine/aspartate aminotrans-ferase increased | 0.000(0.000-) | 0.999 | — | — |
| High blood pressure | 0.000(0.000-) | 1.000 | — | — |
| Vomiting | 0.000(0.000-) | 0.999 | — | — |
| Pruritus | 0.000(0.000-) | 0.999 | — | — |
| Myalgia | 1.536(0.149-15.821) | 0.718 | — | — |
| Weight loss | 0.000(0.000-) | 0.999 | — | — |
| WBC count decrease | 0.000(0.000-) | 1.000 | — | — |
| Arthralgia | 1.301(0.215-7.873) | 0.775 | — | — |
| Hand-foot syndrome | 0.000(0.000-) | 0.999 | — | — |
| Hemorrhage | 0.000(0.000-) | 1.000 | — | — |

TABLE S3. Univariable and multivariable Cox regression of irAE for PFS

|  | Univariable | | Multivariable | |
| --- | --- | --- | --- | --- |
|  | HR(95%CI) | P value | HR(95%CI) | P value |
| Fatigue | 1.073(0.568-2.030) | 0.827 | — | — |
| Fever | 1.127(0.573-2.217) | 0.729 | — | — |
| Rash | 0.453(0.219-0.937) | 0.033 | — | — |
| Vitiligo | 0.537(0.262-1.100) | 0.089 | — | — |
| Decreased appetite | 1.181(0.614-2.272) | 0.618 | — | — |
| Hypothyroidism | 0.706(0.163-3.055) | 0.641 | — | — |
| Oral ulcer | 0.739(0.331-1.648) | 0.460 | — | — |
| Alanine/aspartate aminotrans-ferase increased | 0.566(0.127-2.518) | 0.455 | — | — |
| high blood pressure | 1.012(0.136-7.539) | 0.991 | — | — |
| Vomiting | 0.716(0.342-1.498) | 0.375 | — | — |
| Pruritus | 0.721(0.313-1.664) | 0.444 | — | — |
| Myalgia | 0.510(0.151-1.717) | 0.277 | — | — |
| Weight loss | 0.771(0.269-2.208) | 0.628 | — | — |
| WBC count decrease | 0.340(0.045-2.560) | 0.295 | — | — |
| Arthralgia | 0.786(0.356-1.737) | 0.552 | — | — |
| Hand-foot syndrome | 0.895(0.271-2.951) | 0.855 | — | — |
| Hemorrhage | 0.457(0.061-3.398) | 0.444 | — | — |

TABLE S4. Univariable and multivariable Cox regression of irAE for OS

|  | Univariable | | Multivariable | |
| --- | --- | --- | --- | --- |
|  | HR(95%CI) | P value | HR(95%CI) | P value |
| Fatigue | 0.953(0.427-2.128) | 0.906 | — | — |
| Fever | 0.402(0.166-0.978) | 0.044 | 0.444(0.181-1.087) | 0.075 |
| Rash | 0.343(0.131-0.895) | 0.029 | 0.374(0.142-0.983) | 0.046 |
| Vitiligo | 0.398(0.158-1.003) | 0.051 | 0.742(0.242-2.275) | 0.602 |
| Decreased appetite | 0.917(0.443-1.898) | 0.816 | — | — |
| Hypothyroidism | 0.807(0.107-6.067) | 0.835 | — | — |
| Oral ulcer | 0.469(0.146-1.501) | 0.202 | — | — |
| Alanine/aspartate aminotrans-ferase increased | 1.002(0.127-7.931) | 0.998 | — | — |
| high blood pressure | 3.741(0.482-29.036) | 0.207 | — | — |
| Vomiting | 1.245(0.631-2.456) | 0.527 | — | — |
| Pruritus | 0.039(0.000-13.542) | 0.278 | — | — |
| Myalgia | 2.184(0.718-6.644) | 0.169 | — | — |
| Weight loss | 0.789(0.231-2.696) | 0.705 | — | — |
| WBC count decrease | 0.048(0.000-18517.713) | 0.643 | — | — |
| Hyperthyroidism | 0.564(0.164-1.937) | 0.363 | — | — |
| Arthralgia | 2.009(0.958-4.215) | 0.065 | — | — |
| Hand-foot syndrome | 0.623(0.139-2.789) | 0.536 | — | — |
| Hemorrhage | 2.183(0.288-16.531) | 0.450 | — | — |
